# A New Risk Score for Patients With Acute Chest Pain and Normal High Sensitivity Troponin

**DOI:** 10.3389/fmed.2021.728339

**Published:** 2022-01-04

**Authors:** Chunpeng Ma, Xiaoli Liu, Lixiang Ma

**Affiliations:** ^1^Department of Cardiology, The First Hospital of Qinhuangdao, Qinhuangdao, China; ^2^Department of Endocrinology, The First Hospital of Qinhuangdao, Qinhuangdao, China

**Keywords:** risk stratification, chest pain, coronary artery disease, high-sensitivity troponin I, emergency department

## Abstract

**Objective:** To investigate a new risk score for patients who suffered from acute chest pain with normal high-sensitivity troponin I (hs-TnI) levels.

**Methods:** In this study, patients with acute chest pain who were admitted to the emergency department (ED) of our hospital had been recruited. Hs-TnI was measured in serum samples drawn on admission to the ED. The end point was the occurrence of major adverse cardiac events (MACE) within 3 months. Predictor variables were selected by logistic regression analysis, and external validity was assessed in this study. Furthermore, validation was performed in an independent cohort, i.e., 352 patients (validation cohort).

**Results:** A total of 724 patients were included in the derivation cohort. The results showed that four predictor variables were significant in the regression analysis—male, a history of chest pain, 60 years of age or older and with three or more coronary artery disease (CAD) risk factors. A total of 105 patients in the validation cohort had serious adverse cardiac events. The validation cohort showed a homogenous pattern with the derivation cohort when patients were stratified by score. The area under the curve (AUC) of the receiver operating characteristic (ROC) in the derivation cohort was 0.80 (95% CI: 0.76–0.83), while in the validation cohort, it was 0.79 (95% CI: 0.75–0.82).

**Conclusion:** A new risk score was developed for acute chest pain patients without known CAD and ST-segment deviation and with normal hs-TnI and may aid MACE risk assessment and patient triage in the ED.

## Introduction

Chest pain is one of the most common symptoms presenting among emergency department (ED) patients (1). In the large and heterogeneous population, however, assessment of acute coronary syndrome (ACS) remains a major clinical challenge. Guidelines suggest using risk scores in the ED for early stratification of patients with acute ischemic chest pain and selecting different treatment strategies for different prognoses (2). Several risk scoring systems, such as the thrombolysis in myocardial infarction (TIMI), the Global Registry of Acute Coronary Events (GRACE), HEART, Sanchis, and Florence scores, have been developed to aid in the risk stratification of patients with suspected or diagnosed ACS (3–8). The TIMI and GRACE scores were developed for patients with ACS, the HEART score was developed for patients with the suspected ACS, the Sanchis score was developed for patients with chest pain, non-ST segment deviation ECG and normal troponin levels (5), and the Florence score was developed for patients with acute chest pain without known coronary artery disease (CAD) and with normal ECG and troponin levels (7). However, the data of the Sanchis and Florence scores were derived from databases of 10 years ago when high-sensitivity troponin (hs-TnI) was not used.

Recently, there was no special risk score for acute chest pain patients without known CAD and ST-segment deviation and with normal hs-TnI level in the ED. Therefore, we conducted this study to investigate a new risk score for patients who suffered from acute chest pain with normal hs-TnI levels.

## Materials and Methods

### Subjects

This was a retrospective cohort study. From January 2019 to June 2019, a total of 724 patients with acute chest pain who were admitted to the ED of an urban academic tertiary hospital in Qinhuangdao (China) had been recruited. In addition, from August 2019 to October 2019, a total of 352 patients were recruited as the validation cohort. Hs-TnI was measured in serum samples drawn on admission to the ED. Troponin I was measured using an hs-TnI immunoassay (Enhanced Accu Troponin I, Beckman-Coulter Inc., Brea, CA, USA) (9). Hs-TnI assay in our study had a 99th percentile concentration of 42 ng/l, with a corresponding coefficient of variation of 8% and a limit of detection of 10 ng/l. This study was conducted in accordance with the Declaration of Helsinki and approved by the ethics committee of our hospital. All participants had signed the informed consent.

### Inclusion and Exclusion Criteria

Inclusion criteria: (1) patients who admitted to the ED due to chest pain (“pain” encompasses not only pain but also symptoms, such as discomfort, pressure, and squeezing) of possible coronary origin; (2) patients who were at least 2 h from the onset of symptoms to arrival at the ED; (3) age was older than 18 years old; and (4) patients who have signed informed consent.

Exclusion criteria: (1) patients who had previous myocardial infarction [MI]; (2) patients who had percutaneous intervention [PCI]; (3) patients who had coronary artery bypass graft [CABG]); (4) patients who had ST-segment deviation (depression ≥ 0.05 mV or elevation ≥ 0.1 mV in two or more contiguous leads); (5) elevated hs-TnI was excluded; (6) patients who suffered from an aortic dissection, pulmonary embolism, arrhythmia, trauma, a terminal illness; (7) patients who were pregnant; (8) patients who were unable or unwilling to provide informed consent; and (9) patients whose data were incomplete.

### Candidate Predictor Variables

Data were collected on arrival to develop the score and were retrieved from the case report form. A total of five baseline characteristics were screened as candidate predictor variables for developing an endpoint event. The sole continuous variable was age, and the three dichotomous variables were gender, three or more CAD risk factors (hypertension, hypercholesterolemia, diabetes mellitus, family history of CAD, current smoking status [<1 month], obesity [body mass index ≥ 30 kg/m^2^]), and ischemic stroke. History of chest pain was the sole rank variable.

The history of chest pain was classified by two investigators into three ranks. The first rank was a slight suspicion of CAD if patients only presented with right-sided chest pain or chest pain that worsened with inhalation or palpation. The second rank was a moderate suspicion of CAD, if the history contained both slightly and highly suspicious elements. The third rank was a high suspicion of CAD, if patients presented only with central- or left-sided chest pain with radiation to throat, jaw, shoulders, back, one or both arms, associated diaphoresis, dyspnea, nausea, and/or vomiting. A third opinion was taken to reach a conclusion if there was disagreement regarding classification.

### The Main End Points

Patients were followed up for 3 months by telephone interview and, if appropriate, by assessment of their hospital record. The end point was the occurrence of major adverse cardiac events (MACE) within 3 months after the initial presentation. The criteria for an MACE included any of the following: acute myocardial infarction (AMI), PCI, CABG, and all-cause death. AMI was defined according to the fourth universal definition of MI (8), PCI was defined as any therapeutic catheter intervention in the coronary arteries, and CABG was defined as any cardiac surgery in which coronary arteries were operated on.

### Statistical Analysis

Data were analyzed using the SPSS statistical package (version 20.0, SPSS Inc., Chicago, IL, USA). The continuous variables of normal distribution were expressed as mean ± SD, the continuous variables of non-normal distribution were expressed as median (interquartile range [IQR]), the categorical variables were expressed as frequency (percentage [%]). Each candidate predictor variable was tested independently using univariate logistic regression analysis. Multivariate stepwise (backward elimination) logistic regression analysis-tested variables were significant at *P* < 0.10 in the univariate analysis. Predictor variables associated with *P* < 0.05 were included as components of the score.

After the development of the multivariate regression model, the regression coefficients were rounded to the nearest whole multiple of the smallest coefficient to obtain a simple and appropriate weighting for each variable. The goodness of fit of the model was assessed using the Hosmer-Lemeshow test. The discriminative power of the model was evaluated using the area under the receiver operating characteristic (ROC) curve. The difference in ROC curves between the derivation and validation cohort was analyzed using a Z-score.

Differences in the event rates for increasing risk score and among groups were analyzed using the chi-square test. Fisher's exact test was used when expected frequencies were less than five. The homogeneity of the derivation and validation cohort was tested by comparing the slope of the increase in the event rates with an increasing score using a least-squares linear regression analysis. A two-sided *P* < 0.05 was considered statistically significant.

## Results

### Baseline Characteristics

**Figure 1:**
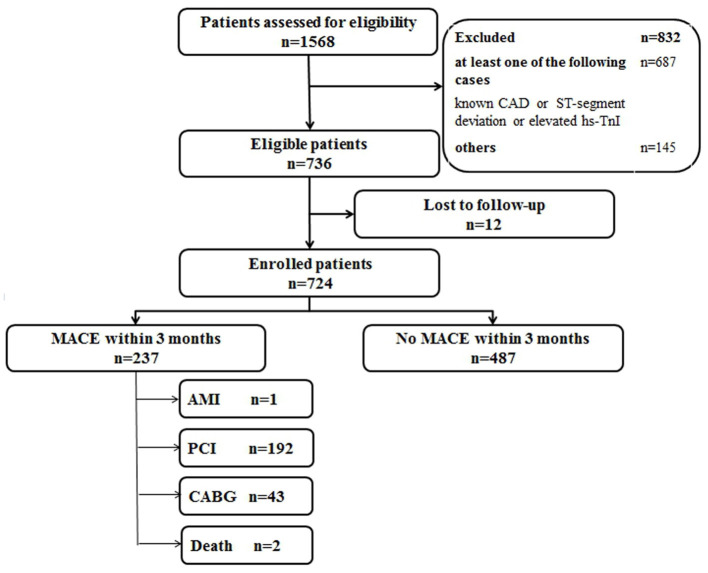
Flow diagram of participants in the derivation cohort study. CAD, coronary artery disease; hs-TnI, high-sensitivity Troponin I; MACE, major adverse coronary events; AMI, acute myocardial infarction; PCI, percutaneous coronary intervention; CABG, coronary artery bypass graft.

A total of 1,568 consecutive chest pain patients presenting to the ED were assessed, and 832 patients were excluded according to the exclusion criteria, leaving 736 patients meeting the criteria necessary for the study. Twelve patients were lost for the 3-month follow-up. Finally, 724 eligible patients were enrolled (Figure 1). The baseline characteristics of the derivation cohort are listed in Table 1.

**Table 1:** Baseline characteristics of the derivation and validation cohort^*^.

**Characteristics**	**Derivation cohort**	**Validation cohort**
	**(*n* = 724)**	**(*n* = 352)**
Age, years (mean ± SD)	58.4 ± 9.8	58.6 ± 9.9
Male sex	417 (57.6%)	200 (56.7%)
History of ischemic chest pain	724 (100%)	352 (100%)
Slight suspicion	96 (13.3%)	63(17.9%)
Moderate suspicion	458 (63.3%)	184(52.3%)
High suspicion	170 (23.5%)	105(29.8%)
Hypertension	441 (60.9%)	190(54.0%)
Hypercholesterolemia	142 (19.6%)	47(13.4%)
Diabetes mellitus	179 (24.7%)	71(20.2%)
Family history of CAD	94 (13.0%)	42(11.9%)
Current smoking	189 (26.1%)	81(23.0%)
Obesity	85 (11.7%)	32(9.1%)
Ischemic stroke	74 (10.2%)	33(9.4%)

*SD, Standard deviation; CAD, coronary artery disease*.

* *Data are N (%) unless otherwise specified*.

### Development of the Score

Age was dichotomized by finding the point of maximum discrimination through analysis of the ROC curves. The area under the ROC ranged between 0.505 and 0.555 for varying age cut-offs in 5-year increments from 40 to 75 years. The area under the ROC was highest at an age cut-off of 60 years, which was close to the median age of 59 years for the study cohort. Therefore, 60 years of age was selected as the cut-off in the final model. Four of the five original candidate predictor variables were statistically significant in the multivariate regression analysis and were included in the final model (see Table 2). The regression coefficient of three or more CAD risk factors was the smallest and its weight was 1 point. The regression coefficients of other variables were rounded to the nearest whole multiple of the smallest coefficient to obtain their weightings (see Table 3). The score was calculated by summing the weightings for the individual elements of the score. The final score ranged from 0 to 9 points. The Hosmer–Lemeshow statistic was 5.570_df8_ (*P* = 0.695). The area under the curve (AUC) of the ROC in the derivation cohort was 0.80 (95% CI: 0.76–0.83). Event rates were increased significantly as the risk score increased (*P* < 0.001 by chi-square for trend).

**Table 2:** Candidate predictor variables of the score in univariate and multivariate logistic regression analysis.

**Candidate predictor variables**	**Univariate analysis**			**Multivariate analysis**		
	**β coefficient**	***P*** **-value**	**OR (95% CI)**	**β coefficient**	***P*** **-value**	**OR (95% CI)**
Age, ≥60 years	0.439	0.006	1.55 (1.14–2.12)	0.661	<0.001	1.94 (1.37–2.74)
Male sex	0.902	<0.001	2.47 (1.77–3.44)	0.958	<0.001	2.61 (1.81–3.74)
History of chest pain						
Slight suspicion	0		1 (reference)	0		1 (reference)
Moderate suspicion	2.685	<0.001	14.65 (4.57–47.04)	2.640	<0.001	14.01 (4.33–45.34)
High suspicion	3.481	<0.001	32.49 (9.90–106.64)	3.427	<0.001	30.79 (9.29–102.12)
3 or more CAD risk factors^*^	0.726	<0.001	2.07 (1.39–3.06)	0.611	0.005	1.84 (1.21–2.82)
Ischemic stroke	0.627	0.012	1.87 (1.15–3.04)	0.500	0.070	1.65 (0.96–2.83)

* *Risk factors included hypertension, hypercholesterolemia, diabetes mellitus, family history of CAD, current smoking, or obesity. OR, odds ratio; CI, confidence interval; CAD, coronary artery disease*.

**Table 3:** Weightings of the predictor variables in the final model.

**Predictor variables**	**Weightings**
Age, ≥60 years	2
Male sex	1
History of chest pain	
Slight suspicion	0
Moderate suspicion	4
High suspicion	5
3 or more CAD risk factors^*^	1

* *Risk factors included hypertension, hypercholesterolemia, diabetes mellitus, family history of CAD, current smoking, or obesity. CAD, coronary artery disease*.

### Validation of the Score

**Figure 2:**
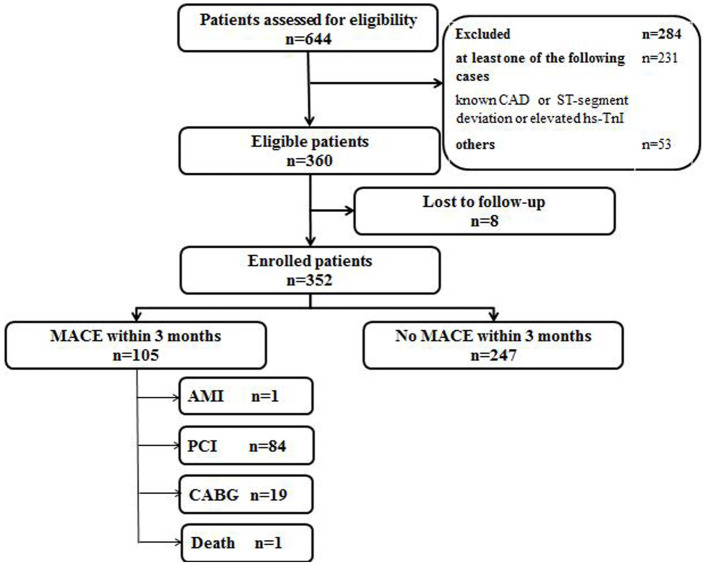
Flow diagram of participants in the validation cohort study. CAD, coronary artery disease; hs-TnI, high-sensitivity Troponin I; MACE, major adverse coronary events; AMI, acute myocardial infarction; PCI, percutaneous coronary intervention; CABG, coronary artery bypass graft.

**Figure 3:**
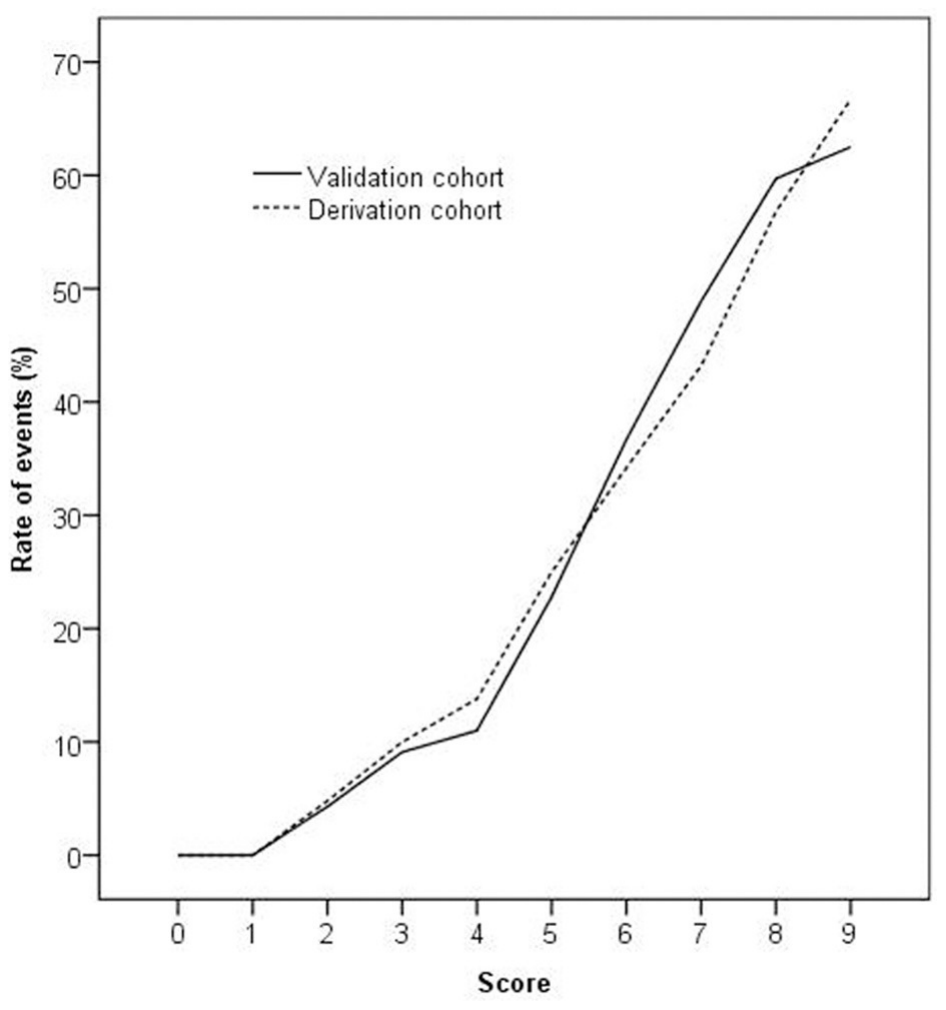
The rate of events increased significantly as the score increased in the derivation and validation cohorts (*P* < 0.001 by chi-square for trend). The slope of the increase in the event rates with an increasing score in the two cohorts was not statistically significant (*P* = 0.879 by least-squares linear regression analysis).

The validation of the score was performed from August 2019 to October 2019. A total of 644 consecutive chest pain patients presenting to the ED were assessed. Total 284 patients were excluded according to exclusion criteria, leaving 360 patients meeting the inclusion criteria. Eight patients were lost to 3 months follow-up. Finally, 352 eligible patients were enrolled, and 105 patients had MACE (Figure 2). The baseline characteristics of the validation cohort are listed in Table 1. One patient was diagnosed with AMI, 84 patients underwent PCI, 19 patients underwent CABG, and 1 was patient died. Event rates increased significantly with increasing scores (*P* < 0.001). The validation cohort showed a homogenous pattern with the derivation cohort when patients were stratified by score as the slope of the increase in the event rates with increasing scores in the two cohorts was not statistically significant (*P* = 0.879; see Figure 3). The AUC of the ROC in the validation cohort was 0.79 (95% CI: 0.75–0.82), which was not significantly different from the derivation cohort (*P* = 0.591).

### Exploring the Utility of the Score

Patients in the derivation cohort were classified into low-, intermediate-, and high-risk groups to explore the utility of the score. The classification was assessed in the validation cohort, the rates of MACE in the three groups were 1.6% (1/63), 19.8% (17/86), and 42.9% (87/203). The rate of derivation cohort MACE was 32.7% (237/724), the rate of MACE in the validation cohort was 29.8% (105/352), and there was no statistical difference in the incidence of MACE between the two groups (see Table 4).

**Table 4:** Classifications of chest pain patients in the derivation cohort and validation cohort.

	**Low risk (0–2)**	**Intermediate risk (3–5)**	**High risk (6–9)**	MACE (rate)^*^
Derivation cohort (*n* = 724)	PCI (1)	PCI (29), CABG (8)	AMI (1), PCI (162), CABG (35), Death (2)	32.7% (237/724)
Validation cohort (*n* = 352)	PCI (1)	PCI (23), CABG (1)	AMI (1), PCI (60), CABG (18), Death (1)	29.8% (105/352)
χ^2^	0.007	0.98	0.388	0.922
*P*–value	0.933	0.323	0.534	0.337
OR (95% CI)	1.13 (0.07–18.39)	1.35 (0.74–2.46)	0.90 (0.64–1.26)	0.87 (0.66–1.15)

* *The rates of MACE in the three classifications in the derivation cohort and the validation cohort were not significantly different (P > 0.05 by χ^2^-test)*.

*MACE, major adverse cardiac events; PCI, percutaneous intervention; CABG, coronary bypass graft; AMI, acute myocardial infarction*.

## Discussion

In this study, a total of 724 patients were included and the outcomes showed that four predictor variables were significant in the regression analysis—male, a history of chest pain, 60 years of age or older, and with three or more CAD risk factors. The History of chest pain, Aged 60 or older, three or more CAD risk factors, and male Sex (HARS) risk score was developed for acute chest pain patients with normal hs-TnI.

Acute chest pain patients without known CAD and ST-segment deviation and with normal troponin levels were probably stratified in the low-risk group according to the guideline (10). However, these patients were heterogeneous and needed to be stratified specifically. The Sanchis score stratified patients into five progressive risk categories in which event rates ranged from 0 to 29.6% (5). The Florence score was able to accurately stratify these patients into three groups in which event rates ranged from 1 to 25% (7). However, one disadvantage of those two scores was that hs-Tn, which is recommended in the evaluation and diagnosis of AMI and prognosis of suspected ACS by guidelines, was unused (11). Another disadvantage was that two complex scores of chest pain symptoms were used, which limited their applicability in the ED (1, 12).

This study specifically developed a new risk score for acute ischemic chest pain patients without known CAD and ST-segment deviation and with normal hs-TnI levels based on logistic regression analysis. The hs-TnI assay in our study had a 99th percentile concentration of 42 ng/l with a corresponding coefficient variation of 8% and a limit of detection of 10 ng/l (13). Four of the five original candidate predictor variables were included in the final model. The most powerful predictor was the history of chest pain, which was consistent with the Florence score (7). However, the history of chest pain was trichotomous in our study, which was simpler than that in the Florence and Sanchis scores and easier to use in the ED. The treatment of age as a continuous variable was inappropriate for the development of a simple risk score; therefore, age was dichotomized. In this score and the Sanchis score, the cut-off for age was selected by finding the point of maximum discrimination through analysis of the ROC curves, which were also used in the TIMI scores (3, 5). In the Florence score, however, the authors did not state how the cut-off for age was selected (7). It is known that some traditional CAD risk factors are weakly predictive of the likelihood of acute cardiac ischemia in the ED and prognostic for event rates in chest pain patients with suspected ACS (2). Therefore, three or more CAD risk factors used as an independent predictor variable in the TIMI score (4) were selected for use in this study and remained statistically significant in the multivariate regression analysis, which was different from Sanchis and Florence scores (5, 7). Finally, male gender was an independent predictor variable in this score, as per the Sanchis and Florence scores (5, 7). The new risk score was termed HARS. The goodness of fit of the final model was excellent, and the discrimination of the risk score was good. The event rates were increased significantly along with the increase in risk scores. The HARS score was validated prospectively, and the discrimination of the risk score in the validation cohort was also good.

To explore the utility of the HARS score in clinical practice, patients were classified into low-, intermediate-, and high-risk groups according to the MACE rate (14–17). However, existing literature uses different boundaries for low-, intermediate-, and high-risk for patients with chest pain (3–7, 18–23). In this study, we defined the boundaries of low, intermediate, and high risk as ≤ 2%, >2% but <20%, and ≥20%, respectively, based on the study population, events, and follow-up time. The MACE rate was significantly different among the three groups and the score may aid patient triage in the ED. In the low-risk group, the MACE rate was only 1.5%, and the two events were selective PCI. Patients in this group could be discharged early from the ED. In the intermediate-risk group, the MACE rate was increased to 17.8%, but no patients had AMI or died. Patients in this group may require further assessment in the ED, such as repeat hs-TnI and ECG testing. In the high-risk group, the MACE rate rose to 44.9%. Patients in this group should be admitted to the hospital and potentially undergo revascularization. The recommendations in our study were very simple and easy to use, which could aid rapid initial triage, reduce crowding, and assign reasonable medical resources in the ED.

In conclusion, the HARS risk score was developed for acute chest pain patients without known CAD and ST-segment deviation and with normal hs-TnI and may aid MACE risk assessment and patient triage in the ED.

### Limitations

Several limitations of this study should be acknowledged. Firstly, some patients with stable or unstable angina could not be excluded, according to our definition of known CAD. Secondly, the prevalence of low-risk and high-risk chest pain is relatively low and high, respectively, in the study population of our ED. Therefore, this risk score will require further assessment in other centers serving patient populations with different disease prevalence. Thirdly, the current analysis only represents a single-center study and the value of the HARS risk score still needs further research.

## Data Availability Statement

The original contributions presented in the study are included in the article/supplementary material, further inquiries can be directed to the corresponding author/s.

## Ethics Statement

The studies involving human participants were reviewed and approved by the Ethics Committee of The First Hospital of Qinhuangdao (No. 2020J005). The patients/participants provided their written informed consent to participate in this study.

## Author Contributions

CM conceived of the study. XL participated in its design and coordination. LM helped to draft the manuscript. All authors read and approved the final manuscript.

## Conflict of Interest

The authors declare that the research was conducted in the absence of any commercial or financial relationships that could be construed as a potential conflict of interest.

## Publisher's Note

All claims expressed in this article are solely those of the authors and do not necessarily represent those of their affiliated organizations, or those of the publisher, the editors and the reviewers. Any product that may be evaluated in this article, or claim that may be made by its manufacturer, is not guaranteed or endorsed by the publisher.
